# An MRI-based study of the insula in a South African population

**DOI:** 10.1007/s00276-024-03458-y

**Published:** 2024-08-09

**Authors:** C. R. Govender, V. Bisetty, N. Naidoo, I. G. Moodley, L. Lazarus

**Affiliations:** 1 Department of Clinical Anatomy, School of Laboratory Medicine and Medical Sciences, University of KwaZulu- Natal (UKZN), Durban, South Africa; 2 College of Medicine and Health Sciences, Mohammed Bin Rashid University of Medicine and Health Sciences (MBRU), Dubai Healthcare City, Dubai United Arab Emirates; 3 Jackpersad and Partners Inc, Specialist Diagnostic Radiologists, Durban, South Africa

**Keywords:** Gyri, Insula, Morphology, Morphometry, MRI, Sulci

## Abstract

**Purpose:**

The insula, a cortical structure buried deep within the sylvian fissure, has long posed a surgical challenge. Comprehensive knowledge of the insular anatomy is therefore integral to preoperative planning and safe interventional procedures. Since magnetic resonance imaging (MRI) is a favoured modality for the identification of cerebral structures, this study aimed to investigate the morphology and morphometry of the insula in a South African population, using MRI scans.

**Methods:**

One-hundred MRI studies of insulae (*n* = 200 hemispheres) were retrospectively analysed for morphological features and morphometric parameters.

**Results:**

The insulae were predominantly trapezoidal in shape (Laterality: Left: 82%; Right: 78%; Sex: Male: 84%, Female: 76%). The central insular sulcus was almost always “well seen” (Laterality: Left: 97%; Right: 99%; Sex: Male: 99%, Female: 97%). The middle short insular gyrus (MSG) was most variable in visibility, especially when compared across the sexes (*p* = 0.004). Insular gyri widths were comparable in both cerebral hemispheres; the posterior long gyrus (PLG) presented with the smallest mean widths. Anterior lobule (AL) widths were larger than those of the posterior lobule (PL). Widths of the insular gyri and lobules were generally larger in males than in females. The MSG and PLG widths in the left hemisphere, AL width in the right hemisphere, and the PL width in both hemispheres were significantly larger in males than in females (*p* = 0.001; *p* = 0.005; *p* = 0.041; *p* = 0.001, *p* = 0.015, respectively).

**Conclusion:**

MRI scans may be used to accurately interpret insular anatomy. The data obtained may aid neurosurgeons to perform safe insula-related surgical procedures.

## Introduction

The human insula is a cortical structure located deep within the lateral sulcus (i.e., sylvian fissure) in each cerebral hemisphere [3, 16]. Due to extensive cerebral development, the insula is covered at birth by parts of the frontal, parietal, and temporal lobes, i.e., the fronto-orbital, frontoparietal, and temporal opercula [11, 16, 18]. Based on its initial description by Johann-Christian Reil in the early 1800s, the insula is also referred to as the “Island of Reil” [14, 19]. It is often considered the fifth lobe of the brain [2, 17].

The insula is pyramidal in shape, comprising three sides that meet at the apex [13]. Two-dimensionally, it is described as being trapezoidal- or triangular-shaped [2, 3, 6, 10, 11]. The insula is anatomically divided into anterior and posterior lobules by the central insular sulcus (CIS), which is the main and deepest sulcus of the insula, coursing obliquely in the postero-superior to antero-inferior direction [13, 18, 19]. The larger anterior lobule (AL) of the insula comprises three short insular gyri viz. the anterior short insular gyrus (ASG), middle short insular gyrus (MSG), and posterior short insular gyrus (PSG), while the smaller posterior lobule (PL) is characterized by two long insular gyri viz. the anterior long insular gyrus (ALG) and posterior long insular gyrus (PLG). However, considerable variation exists in the anatomical features of the insula [1–4, 13]. In particular, intra- and inter-individual differences have been reported in the number of sulci and gyri between left and right cerebral hemispheres [3, 19].

The insula is both anatomically and functionally complex. Due to its concealed location, the insula is not as well understood as other cortical areas [11]. Since the primary gustatory cortex, insular language area, and significant vestibular integration centres are located within the insula, it is thought to play a vital role in taste perception, cognition, motor speech integration, language comprehension, cardiovascular regulation, as well as somatosensory and viscerosensory control [11, 13, 14]. The insula has also been related to learning disabilities and mental health illnesses [9].

Over past decades, the insula has become a major region of interest due to its implication in various neurological and psychiatric disorders [9, 14, 16, 17]. It continues to attract interest, through the aid of modern neuroimaging modalities, because of the growing need for safer intra-operative exploration [2, 13]. As magnetic resonance imaging (MRI) is deemed to be the modality of choice for the examination of soft tissue structures [8], the insula is easily identifiable on MRI studies of the brain [13]. Sound knowledge of the insular anatomy is essential for both functional MRI and diagnosis [13]. The significant functional role of the insula further necessitates a thorough understanding of the insular anatomy, particularly for neurosurgeons [11]. Several studies have reported on the gross anatomy of the insula [2, 3, 10, 13, 19]; however, few MRI studies investigated the insular anatomy and its variations [1, 3, 11, 13]. We conducted the first two-dimensional (2D) MRI study of the insular anatomy in the South African population. Through this study, we aim to investigate the morphology and morphometry of the insula in the relevant population.

## Materials and methods

### Study design and landscape

This retrospective study was conducted at a private medical institution in the Durban metropolitan area, KwaZulu Natal, South Africa. Ethical approval was obtained from the Biomedical Research Ethics Committee (BREC) of the University of KwaZulu-Natal (Reference number: BREC/00004502/2022). The data set comprised of 100 T1- and T2-weighted sagittal section 2D MRI studies (1–5 mm slice thickness), providing 200 right and left cerebral hemispheres (*n* = 200; male: *n* = 94, female: *n* = 106) for image analysis. These studies were acquired using the 1.5-T clinical MR systems (Signa; GE Medical Systems, Milwaukee, WI). Sagittal MRI scans were used as they are appropriate for visualisation and evaluation of the insular gyri [3]. MRI series were excluded if they displayed any type of pathological condition or were of poor imaging quality. The MRIs were reviewed and analysed using the RadiAnt DICOM Viewer software (Mexidant, 2011).

### Anatomical evaluation

This study entailed evaluation of the morphological features and morphometric parameters of the insula.

### Morphology of the insula

**Fig. 1:**
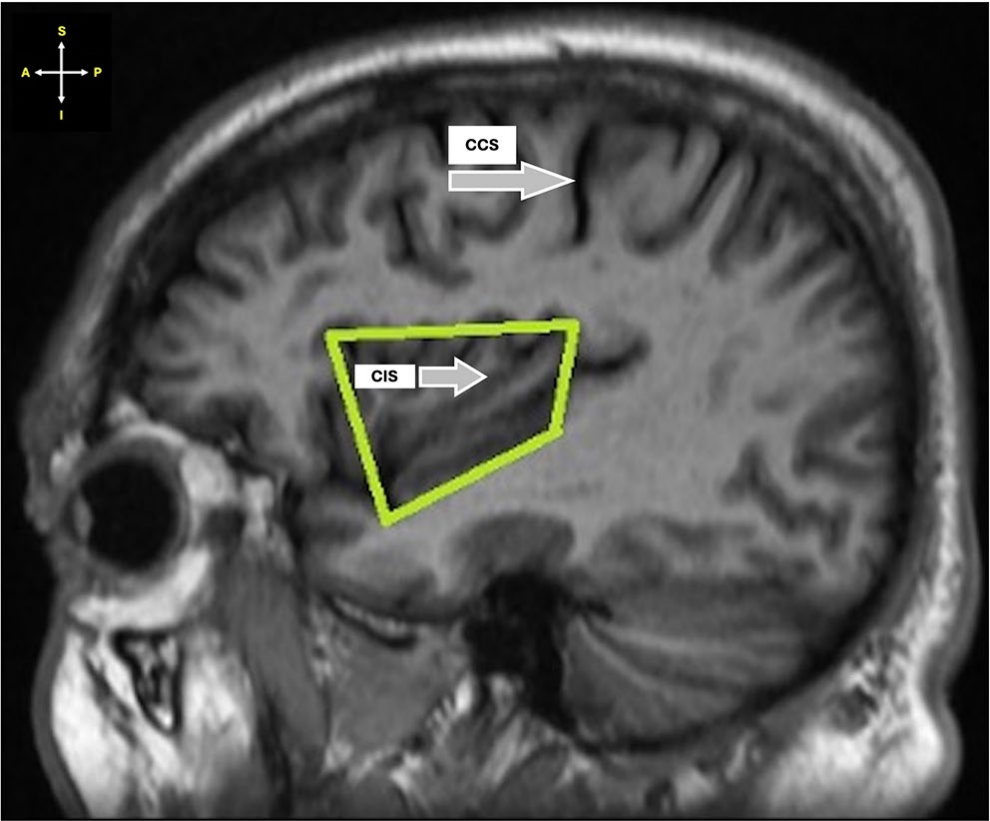
Sagittal section of MRI showing trapezoidal-shaped insula. *Key* CCS – Central cerebral sulcus; CIS – Central insular sulcus

**Fig. 2:**
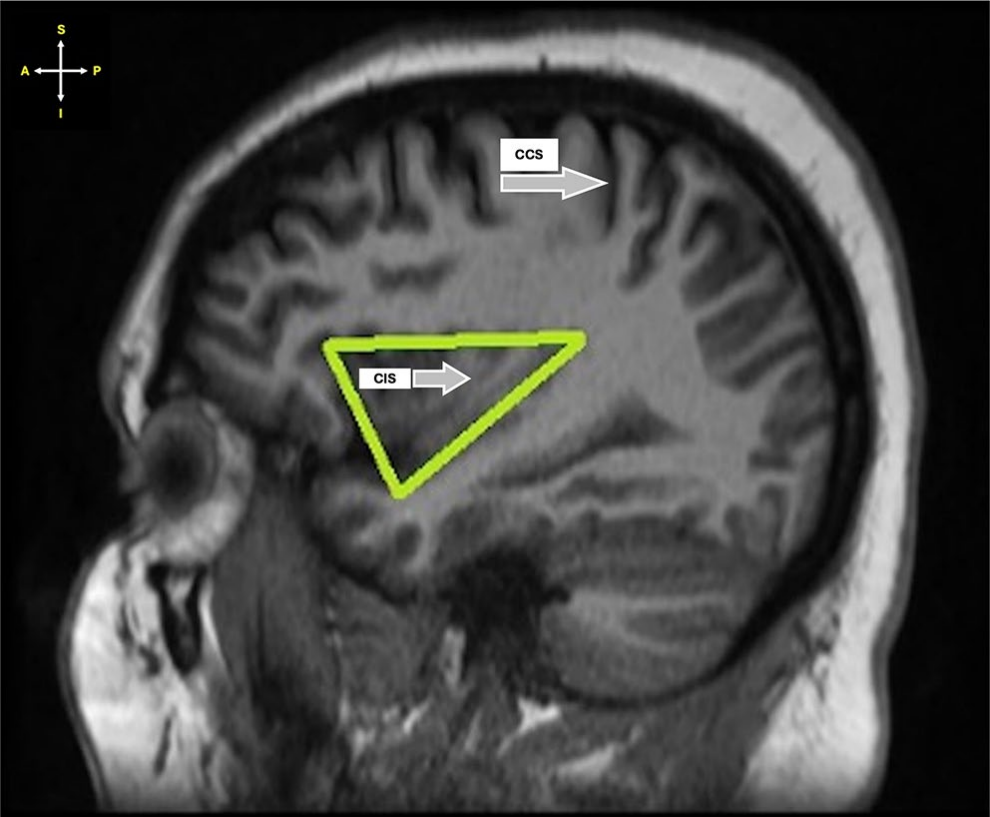
Sagittal section of MRI showing triangular-shaped insula. *Key* CCS – Central cerebral sulcus; CIS – Central insular sulcus

**Fig. 3:**
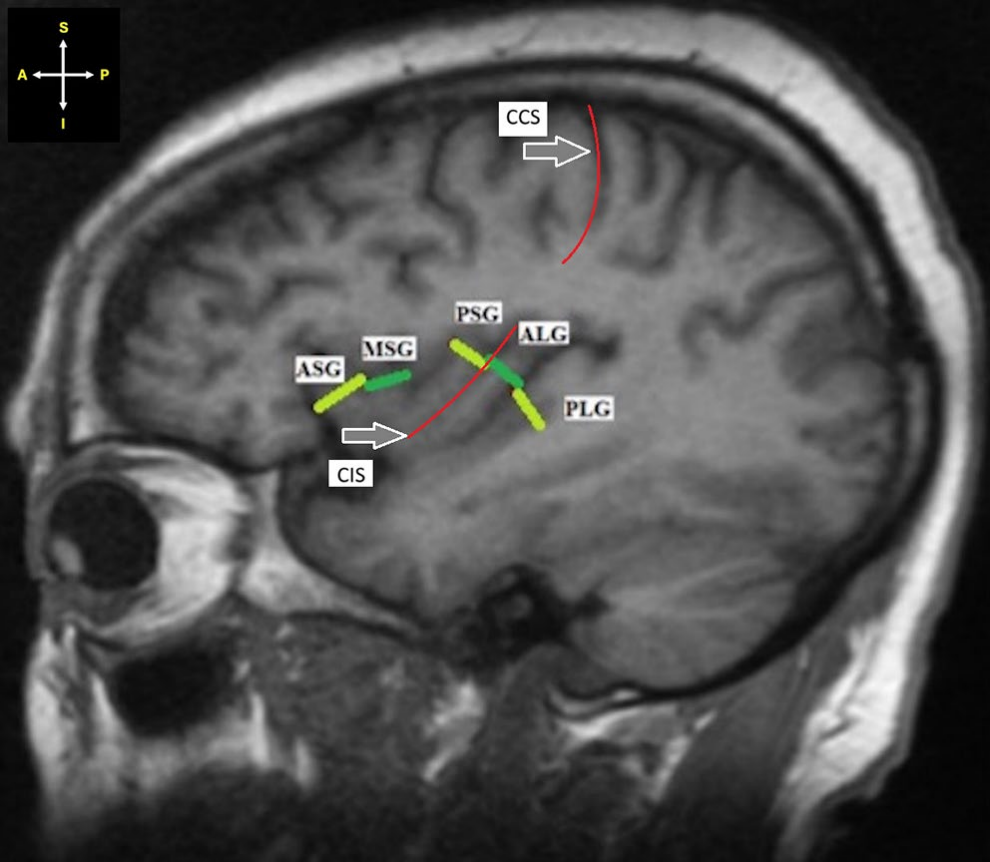
Sagittal section of MRI showing the location of the insular gyri in relation to the CIS. *Key* CCS – Central cerebral sulcus; CIS – Central insular sulcus; ASG -Anterior short insular gyrus; MSG - Middle short insular gyrus; PSG - Posterior short insular gyrus; ALG - Anterior long insular gyrus; PLG - Posterior long insular gyrus

The following morphological features of the insula were documented:

i. The shape of the insula was classified as either trapezoidal or triangular (Figs. 1 and 2).

ii. The insular sulcus in-line with the central cerebral sulcus (CCS) was identified as the CIS. The ASG, MSG and PSG were identified as the gyri anterior to the CIS, while the ALG and PLG were identified as the gyri posterior to the CIS (Fig. 3). The visibility of the gyri and CIS were then graded according to the scale used by Naidich et al. [13] as “not seen”, “poorly seen”, and “well seen”.

### Morphometry of the insula

The following morphometric parameters of the insula were measured only on “well-seen” structures using the built-in RadiAnt measurement tool:

#### Width of the insular gyri

**Fig. 4:**
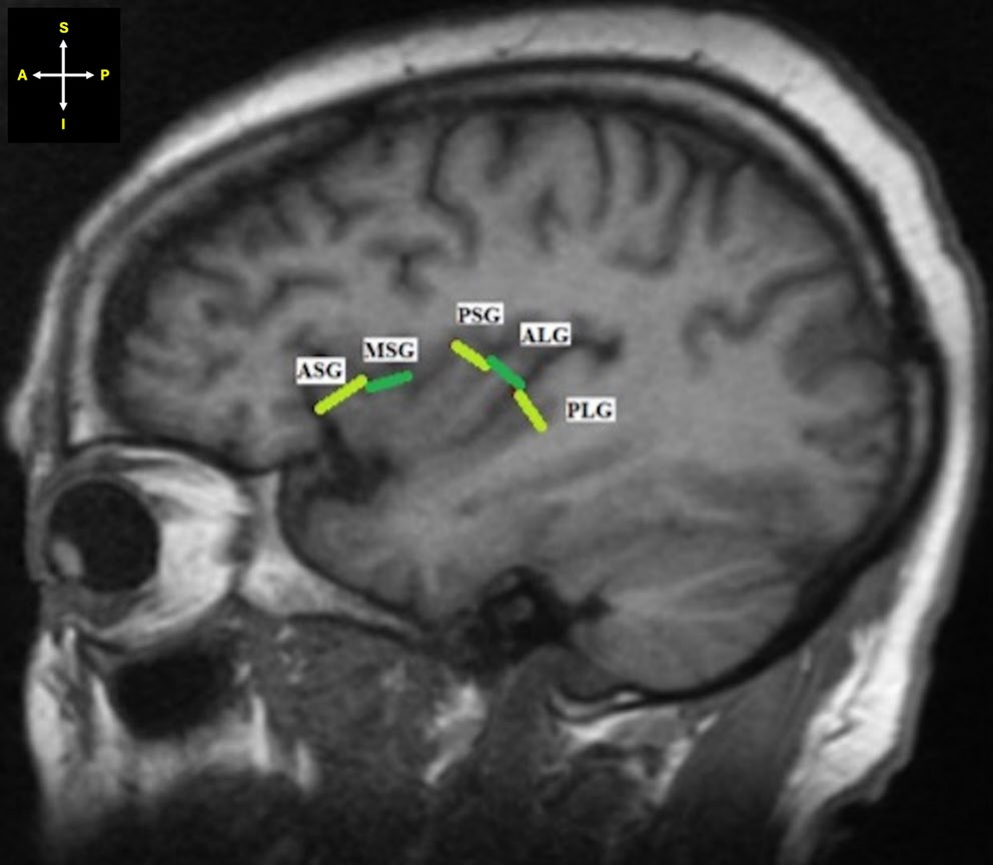
Sagittal section of MRI showing the maximum width of each gyrus. *Key* ASG -Anterior short insular gyrus; MSG - Middle short insular gyrus; PSG - Posterior short insular gyrus; ALG - Anterior long insular gyrus; PLG - Posterior long insular gyrus

The maximum width of each gyrus was measured from the anterior-most point of a gyrus to the posterior-most point of the respective gyrus (Fig. 4).

#### Width of the insular lobules

**Fig. 5:**
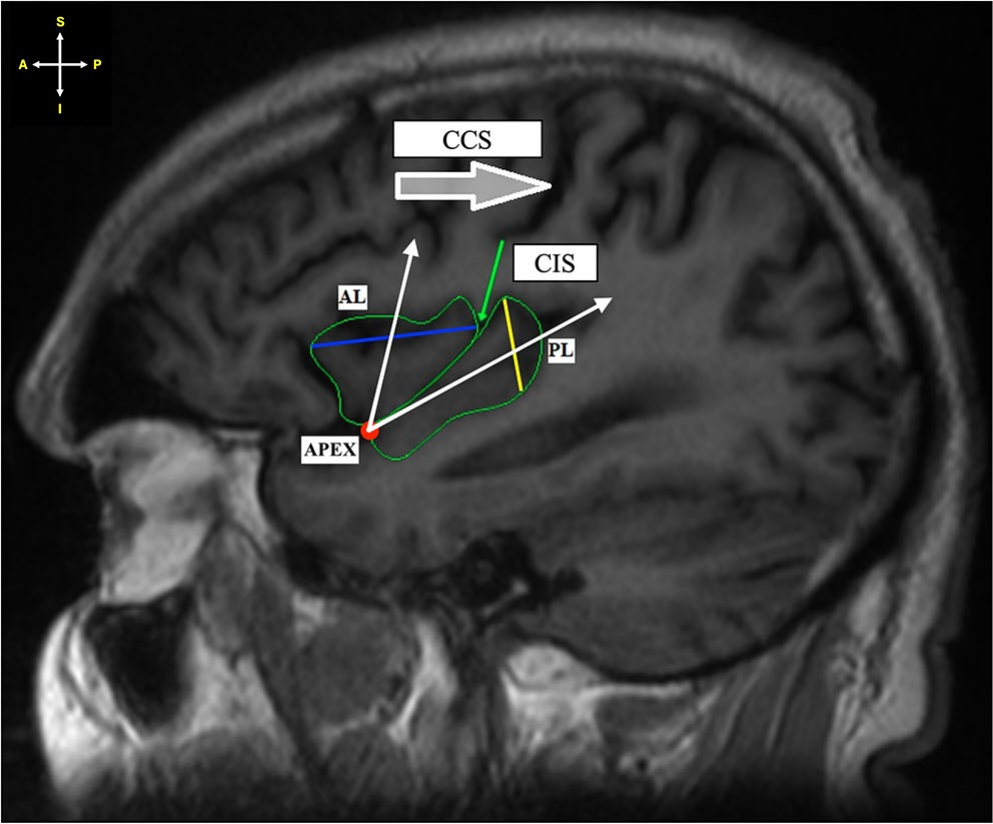
Sagittal section of MRI showing the maximum width of each lobule. *Key* AL - Anterior lobule; PL - Posterior lobule; CCS - Central cerebral sulcus; CIS - Central insular sulcus. The white lines represent the directional axis of each lobule

Due to the topographical anatomy of the insula, the gyri of the AL project in a superior fashion from the apex of the insula (which is directed anterobasally), while the gyri of the PL project more posterosuperiorly. As a result, the authors are of the opinion that it is more appropriate to measure the width of the AL from the anterior-most point of the lobule to the posterior-most point of the respective lobule and the width of the PL from the anterosuperior-most point of the lobule to the posteroinferior-most point of the respective lobule. So, in essence, the maximum width of the base of each lobule is documented (Fig. 5).

### Statistical analysis

Descriptive statistics are reported. Statistical data analysis was performed using the Statistical Package for Social Sciences software (SPSS IBM, Version 24.0). Continuous variables (widths) were screened for extreme values using Histogram and Box plots. To ensure reliability, each parameter was measured three times by a single observer. The intraclass correlation coefficient (ICC) between the three measures was computed. Differences between left and right sides of the insular gyri and lobule widths were analysed using the paired t-test. Comparisons between male and female morphometry parameters were made using the t-test. For morphological parameters, the Chi-Square test was used to compare differences in shape and degrees of visibility in terms of sex and laterality. Agreement between the left and right sides of the morphology parameters were determined using Prevalence Adjusted and Bias Adjusted Kappa (PABAK) with a 95% Confidence Interval (CI) [5]. If the morphology parameters had 3 or more categories, then the Weighted Kappa was used. Significance was set at *p* < 0.05 level.

## Results

### Morphological features of the insula

#### Shape of the insula

Overall, trapezoidal-shaped insulae were reported in 84% of males and 76% of females, while triangular-shaped insulae were reported in 16% of males and 24% of females, respectively. With regards to laterality, the insula was found to be trapezoidal-shaped in 82% of the left hemispheres and 78% of the right hemispheres, and triangular-shaped in 18% of the left and 22% of the right hemispheres. The shape of the insula was independent of sex and laterality (*p* = 0.157; *p* = 0.595) (Table 1).

**Table 1:** Frequency in insular shape and visibility between the right and left cerebral hemispheres in males and females

Morphological feature			Sex (%)			Side of cerebral hemisphere (%)		
			Male (*n* = 94)	Female (*n* = 106)	*p*-value	Left (*n* = 100)	Right (*n* = 100)	*p*-value
Shape		Trapezoidal	84	76	Chisq = 2.00 *p* = 0.157	82	78	Chiq = 0.281 *p* = 0.595
		Triangular	16	24		18	22	
Sulci and Gyri Visibility	**CIS**	**Well-seen**	99	97	Chisq = 1.02 *p* = 0.312	97	99	chisq = 0.255 *p* = 0.613
		Poorly seen	1	3		3	1	
		Not seen	0	0		0	0	
	ASG	Well-seen	96	98	Chisq = 0.687 *p* = 0.407	95	99	Chisq = 1.546 *p* = 0.950
		Poorly seen	4	2		5	1	
		Not seen	0	0		0	0	
	MSG	**Well-seen**	72	59	Chisq = 10.669 *p* = 0.004	66	64	Chisq = 0.102 *p* = 0.950
		Poorly seen	27	29		27	29	
		Not seen	1	12		7	7	
	**PSG**	Well-seen	98	99	Chisq = 0.338 *p* = 0.560	98	99	Chisq = 0.00 *p* = 1.000
		Poorly seen	2	1		2	1	
		Not seen	0	0		0	0	
	ALG	Well-seen	99	99	Chisq = 0.0 *p* = 1.000	98	100	Chisq = 0.505 *p* = 0.477
		Poorly seen	1	1		2	0	
		Not seen	0	0		0	0	
	PLG	Well-seen	50	45	Chisq = 0.506 *p* = 0.776	52	43	Chisq = 0.353 *p* = 0.552
		Poorly seen	49	54		47	56	
		Not seen	1	1		1	1	

*Key* Central insular sulcus (CIS), anterior short insular gyrus (ASG), middle short insular gyrus (MSG), posterior short insular gyrus (PSG), anterior long insular gyrus (ALG), posterior long insular gyrus (PLG) and Chi-square test (Chisq)

#### Visibility of central insular sulcus and insular gyri

In males, 1% of the CIS were “poorly seen” and 99% were “well seen”. The CIS in females were noted to be “poorly seen” (3%) and “well seen” (97%). In the left cerebral hemispheres, 3% of the CIS were “poorly seen” and 97% were “well seen”, while the right cerebral hemispheres presented with 1% “poorly seen” and 99% “well seen” CIS. Visibility of the CIS was not dependant on sex and laterality (*p* = 0.312; *p* = 0.613) (Table 1).

In males, 4% of the ASG were “poorly seen” and 96% were “well seen”, while females presented with 2% of ASG which were “poorly seen” and 98% “well seen” ASG. In the left cerebral hemispheres, 5% of the ASG were “poorly seen” and 95% were “well seen”, whereas in the right cerebral hemispheres, only 1% of the ASG were “poorly seen” and 99% were “well seen”. Visibility of the ASG was not dependant on sex and laterality (*p* = 0.407; *p* = 0.950) (Table 1).

In males, 1% of the MSG were “not seen”, 27% were “poorly seen”, and 72% were “well seen”. In females, 12% of the MSG were “not seen”, 29% were “poorly seen”, and 59% were “well seen”. A total of 7% of the MSG were “not seen” in the left cerebral hemispheres, while 27% and 66% of the MSG were noted to be “poorly seen” and “well seen”, respectively. In the right cerebral hemispheres, the appearance of the MSG was documented as “not seen” (7%), “poorly seen” (29%) and “well seen” (64%). Visibility of the MSG was dependant on sex but not on laterality (*p* = 0.004; *p* = 0.950) (Table 1).

The PSG in males was “poorly seen” (2%) and “well seen” (98%), whilst the PSG in females was “poorly seen” (1%) and “well seen” (99%). In the left cerebral hemispheres, 2% of the PSG were “poorly seen” and 98% were “well seen”, while in the right cerebral hemispheres, only 1% was “poorly seen” and 99% were “well seen”. Visibility of the PSG was not dependant on sex and laterality (*p* = 0.560; *p* = 1.000) (Table 1).

In both males and females, 1% of ALG were “poorly seen” and 99% were “well seen”. The left cerebral hemispheres displayed 2% “poorly seen” ALG, and 98% “well seen” ALG, while 100% of the ALG in the right cerebral hemispheres were “well seen”. Visibility of the ALG was not dependant on sex and laterality (*p* = 1.000; *p* = 0.477) (Table 1).

In males, the PLG was recorded to be “not seen” (1%), “poorly seen” (49%) and “well seen” (50%), while females presented with PLG that were “not seen” (1%), “poorly seen” (54%), and “well seen” (45%). The left cerebral hemispheres presented with 1% “not seen” PLG, 47% “poorly seen” PLG, and 52% “well seen” PLG. In the right cerebral hemispheres, 1% of PLG were “not seen”, 56% “poorly seen”, and 43% were “well seen”. Visibility of the PLG was not dependant on sex and laterality (*p* = 0.776; *p* = 0.552) (Table 1).

### Morphometric parameters of the insula

#### Width of the gyri

The mean widths of the ASG were 7.49 ± 1.29 mm and 7.42 ± 1.23 mm in the left and right cerebral hemispheres, respectively (*p* = 0.678). The mean width of the ASG in the left cerebral hemispheres was 7.60 ± 1.30 mm in males and 7.40 ± 1.30 mm in females (*p* = 0.352), while the mean width of the ASG in the right cerebral hemispheres was 7.50 ± 1.10 mm in males and 7.30 ± 1.40 mm in females (*p* = 0.422) (Table 2).

The MSG had mean widths of 6.79 ± 1.29 mm and 6.95 ± 1.37 mm in the left and right cerebral hemispheres, respectively (*p* = 0.419). The mean width of the MSG in the left cerebral hemispheres was 7.20 ± 1.10 mm in males and 6.30 ± 1.5 mm in females (*p* = 0.001), while the right cerebral hemispheres had a mean MSG width of 7.20 ± 1.30 mm in males and 6.70 ± 1.40 mm in females (*p* = 0.119) (Table 2).

The mean widths of the PSG were 7.45 ± 1.38 mm and 7.66 ± 1.43 mm in left and right hemispheres, respectively (*p* = 0.202). In the left cerebral hemispheres, the mean width of the PSG was 7.50 ± 1.50 mm and 7.40 ± 1.30 mm in males and females, respectively (*p* = 0.873). In the right cerebral hemispheres, the mean PSG widths were 7.90 ± 1.20 mm and 7.40 ± 1.60 mm in males and females, respectively (*p* = 0.072) (Table 2).

The mean width of the ALG was 7.41 ± 1.39 mm in the left cerebral hemisphere and 7.08 ± 1.29 mm in the right cerebral hemisphere (*p* = 0.061). In the left cerebral hemispheres, mean ALG widths of 7.70 ± 1.60 mm and 7.20 ± 1.20 mm were recorded in males and females, respectively (*p* = 0.067). In the right cerebral hemispheres, males had a mean ALG width of 7.20 ± 1.00 mm, and females had a mean ALG width of 7.00 ± 1.50 mm (*p* = 0.297) (Table 2).

The mean PLG widths of 6.00 ± 1.06 mm and 5.91 ± 0.97 mm were recorded for the left and right cerebral hemispheres, respectively (*p* = 0.474). In the left cerebral hemispheres, the mean PLG width was significantly larger in males (6.30 ± 1.10 mm) than in females (5.70 ± 1.00 mm) (*p* = 0.005). In the right cerebral hemispheres, the mean PLG width was 6.10 ± 0.90 mm in males and 5.8 ± 1.00 mm in females (*p* = 0.090) (Table 2).

#### Width of the AL and PL

The mean widths of the AL in the left and right cerebral hemispheres were 29.36 ± 3.82 mm and 29.33 ± 3.74 mm, respectively (*p* = 0.926). In the left cerebral hemispheres, the mean AL width was 30.00 ± 3.50 mm in males and 28.80 ± 4.00 mm in females (*p* = 0.127). In the right cerebral hemispheres, the mean AL width was significantly larger in males (30.10 ± 3.40 mm) than in females (28.60 ± 3.90 mm) (*p* = 0.041) (Table 2).

The PL presented with mean widths of 15.40 ± 2.64 mm in the left cerebral hemisphere and 15.20 ± 2.00 mm in the right cerebral hemisphere (*p* = 0.481). In the left cerebral hemispheres, the mean width of the PL was significantly larger in males (16.30 ± 2.00 mm) than in females (14.6 ± 2.90 mm) (*p* = 0.001). In the right cerebral hemispheres, the mean PL width was significantly larger in males (15.70 ± 1.90 mm) than in females (14.80 ± 2.00 mm) (*p* = 0.015) (Table 2).

**Table 2:** Mean widths of the insular gyri and lobules between the right and left cerebral hemispheres in males and females

Insular gyri and lobule	Width (mm)								
	Left hemisphere [*n* = 100]	Right hemisphere [*n* = 100]	*p*-value	Left hemisphere [*n* = 100]			Right hemisphere [*n* = 100]		
				Male (SD) [*n* = 47]	Female (SD) [*n* = 53]	*p*-value	Male (SD) [*n* = 47]	Female (SD) [*n* = 53]	*p*-value
ASG	7.49 ± 1.29	7.42 ± 1.23	0.678	7.60 ± 1.30	7.40 ± 1.30	0.352	7.50 ± 1.10	7.30 ± 1.40	0.422
MSG	6.79 ± 1.36^a^	6.95 ± 1.37^a^	0.419	7.20 ± 1.10	6.30 ± 1.5^c^	**0.001**	7.20 ± 1.30^c^	6.70 ± 1.40^d^	0.119
PSG	7.45 ± 1.38	7.66 ± 1.43	0.202	7.50 ± 1.50	7.40 ± 1.30	0.873	7.90 ± 1.20	7.40 ± 1.60	0.072
ALG	7.41 ± 1.39	7.08 ± 1.29	0.061	7.70 ± 1.60	7.20 ± 1.20	0.067	7.20 ± 1.00	7.00 ± 1.5	0.297
PLG	6.00 ± 1.06	5.91 ± 0.97	0.474	6.30 ± 1.10	5.70 ± 1.00^e^	**0.005**	6.10 ± 0.90^c^	5.8 ± 1.00	0.090
AL	29.36 ± 3.82^b^	29.33 ± 3.74^b^	0.926	30.00 ± 3.50	28.80 ± 4.00	0.127	30.10 ± 3.40	28.60 ± 3.90	**0.041**
PL	15.40 ± 2.64	15.20 ± 2.00	0.481	16.30 ± 2.00	14.6 ± 2.90	**0.001**	15.70 ± 1.90	14.80 ± 2.00	**0.015**

*Key* Anterior short insular gyrus (ASG), middle short insular gyrus (MSG), posterior short insular gyrus (PSG), anterior long insular gyrus (ALG) and posterior long insular gyrus (PLG). N.B. a: *n* = 90; b: *n* = 98; c: *n* = 46; d: *n* = 47; e: *n* = 52

### Intra-observer reliability and agreement

**Table 3:** Intra-observer agreement between left and right insular morphology parameters

Morphological feature	PABAK/ weighted kappa	95% CI
Shape	PABAK = 0.68	0.50–0.81
CIS visibility	PABAK = 0.96	0.85–0.99
ASG visibility	PABAK = 0.88	0.74–0.95
MSG visibility	Weighted Kappa = 0.453	0.23–0.67
PSG visibility	PABAK = 0.94	0.83–0.98
ALG visibility	PABAK = 0.96	0.85–0.99
PLG visibility	Weighted Kappa = 0.31	0.13–0.50

*Key* Prevalence-adjusted and Bias-adjusted Kappa (PABAK), Central insular sulcus (CIS), anterior short insular gyrus (ASG), middle short insular gyrus (MSG), posterior short insular gyrus (PSG), anterior long insular gyrus (ALG) and posterior long insular gyrus (PLG)

**Table 4:** Intraclass correlation coefficients (ICC) for intra-observer reliability of morphometry parameters

Insular gyri and lobule	ICC	95% CI
Left ASG	0.973	0.94–0.98
Right ASG	0.930	0.85–0.97
Left MSG	0.970	0.93–0.98
Right MSG	0.939	0.86–0.97
Left PSG	0.916	0.82–0.96
Right PSG	0.929	0.85–0.97
Left ALG	0.979	0.95–0.99
Right ALG	0.911	0.81–0.96
Left PLG	0.927	0.84–0.96
Right PLG	0.835	0.65–0.93
Left AL	0.957	0.91–0.98
Right AL	0.987	0.97–0.99
Left PL	0.960	0.91–0.98
Right PL	0.882	0.75–0.95

*Key* Intraclass correlation coefficients (ICC), confidence interval (CI), anterior short insular gyrus (ASG), middle short insular gyrus (MSG), posterior short insular gyrus (PSG), anterior long insular gyrus (ALG) and posterior long insular gyrus (PLG)

## Discussion

The insula has long been an overlooked cerebral structure but is recently gaining attention due to its underlying involvement in neurological and psychological disorders [9, 14, 16]. Literature on the anatomy of the insula is scarce, with only a few studies having analysed its morphology and morphometry using radiological modalities [1, 3, 11, 13].

### Morphological features

#### Shape of the insula

In the current study, insulae predominantly resembled a trapezoidal shape in both right and left cerebral hemispheres (Table 1). A study by Cunha Cabral et al. [6] also found insular shape to vary between trapezoidal and triangular, however, their study reported primarily triangular shaped insulae. Studies by Afif et al. [1], Afif and Mertens [2], and Atlasi *et* al. [3] describe the insula as being trapezoidal in shape. While studies by Guenot *et* al. [10] and Mavridis et al. [11] describe the insula as having a triangular shape. The present study also noted that the insula was predominantly trapezoidal in both males and females. These findings suggest that insular shape is not dependant on sex or laterality (Table 1). The current study is the first to report on shape in terms of sex and laterality.

#### Visibility of central insular sulcus and insula gyri

The CIS was “well seen” in the majority of left and right cerebral hemispheres (Table 1). These values were distinctly higher compared to those obtained by Naidich et al. [13]. The CIS was also mostly “well seen” in both males and females (Table 1). It may be postulated that the CIS is a reliable anatomical landmark on sagittal T1- and T2-weighted MRIs to identify major insular sulci and gyri. Additionally, the CIS was noted to lie in continuity with the central cerebral sulcus, as described in the literature [4].

The ASG and PSG were nearly always well displayed in the left and right cerebral hemispheres (Table 1). These results compared favourably with those of Naidich et al. [13] and Atlasi et al. [3]. The ASG and PSG were also predominantly “well seen” in males and females, corroborating the studies of Cunha-Cabral et al. [6] and Wysiadecki et al. [20] who reported the ASG and PSG to be “well developed” in their cadaveric studies. This suggests that MRI scans are a reliable modality in which to view the insular gyri.

Amongst the short gyri, the MSG presented less frequently and displayed more variable visibility than other gyri. This difference was significant for sex (*p* = 0.004) (Table 1), which may be due to female insulae being typically smaller than male insulae [11]. The MSG was “not seen” in only a few cerebral hemispheres, concurring with the findings of Naidich et al. [13], but in contrast to those of Atlasi et al. [3]. In this study, the MSG was “well seen” in 66% of left insulae and 64% of right insulae, the frequencies of which were lower than the report of Naidich et al. [13]. On the contrary, cadaveric studies by Cunha-Cabral et al. [6] and Wysiadecki et al. [20] recorded the MSG to be “well developed” only in 41.7% and 50% of insulae, respectively. In addition, the MSG was “not seen” in very few male and female insulae of the present study (Table 1).

The current study found that the ALG was “well seen” far more often than the PLG for both cerebral hemispheres (Table 1), thereby corroborating the findings of Naidich et al. [13]. However, this differs from Atlasi et al. [3] who found that both the ALG and PLG were predominantly “well seen” in both cerebral hemispheres. The ALG was also “well seen” in the majority of male and female insulae (Table 1). These findings were supported by Cunha-Cabral et al. [6] and Wysiadecki et al. [20]. On the other hand, the PLG was only “well seen” in 50% or less in both males and females. Cunha-Cabral et al. [6] reported that 86.4% of PLG were “well developed” which does not reflect the results of this study. Visibility of the CIS, ASG, PSG, ALG and PLG was independent of sex and laterality (Table 1). The insular gyri pattern identified in this study concurs that described in the literature [1–3, 13].

### Morphometric parameters of the insula

#### Width of the gyri

The mean widths of the ASG, MSG, PSG, ALG, and PLG were comparable in the left and right cerebral hemispheres, thus corroborating the cadaveric study by Cunha-Cabral et al. [6] (Table 2). The mean widths of all five gyri were larger in males than in females for both cerebral hemispheres (Table 2). These findings align with those of Atlasi et al. [3], who found that the mean antero-posterior distance of the insular base was larger in males than in females for both cerebral hemispheres. Mavridis et al. [11] noted a similar finding and stated that this was “more or less expected” as males tend to have larger and heavier brains.

Of all the insular gyri, the PLG was found to have the smallest mean widths in both cerebral hemispheres, as well as in both sexes (Table 2). In contrast, the ASG and PSG presented with similarly larger mean widths in both right and left cerebral hemispheres and in males and females alike (Table 2). The large variability in MSG and PLG visibility could be a result of their much shorter widths, relative to the other insular gyri. The largely consistent appearance of the ASG, PSG, and ALG may be attributed to their longer widths. This may be useful in a clinical setting, particularly for pre-planning of surgical procedures within the insular region. A study by Shura et al. [16] investigated the structural connectivity of the insula with regard to functionality and noted that the MSG contains a division that separates the anterior and posterior functional subdivisions. This may be a contributing factor to the variation in visibility and width of the MSG.

Statistically significant p-values were yielded for the MSG and PLG in the left cerebral hemispheres between males and females, owing to the distinctly larger morphometric parameters in males (Table 2).

#### Width of the AL and PL

The mean width of the AL was markedly larger than that of the PL (Table 2). Previous studies on non-human primates have shown that the anterior region of the insula (ASG and anterior ventral insular area) is primarily associated with the inferior frontal, orbitofrontal, and anterior temporal cortices and has connections with the amygdala, ventral striatum as well as the cingulate, entorhinal, and adjacent periamygdaloid temporal cortices, while the posterior region of the insula (PLG and posterior ventral insular area) is primarily associated with the temporal cortices and has connections to the dorsal striatum and the adjacent parietal and somatosensory cortices, the supplementary motor area, as well as the primary vestibular cortex [7, 16]. The anterior insula is thereby associated with olfactory, gustatory, viscero-autonomic, homeostatic, and limbic functions, whereas the posterior insula is linked to somatosensory, skeletomotor, auditory and vestibular function [7, 12, 15]. Moreover, Phan et al. [15] reported that the anterior lobule was stimulated by feelings of happiness, sadness, fear, anger, and disgust, while the posterior lobule was only stimulated by fear. The larger width of the AL in this study may be attributed to the numerous functional areas present within the anterior insula. The mean widths of both the AL and PL were also relatively comparable within their respective right and left cerebral hemispheres (Table 2). However, the AL and PL widths were larger in males than in females for both cerebral hemispheres (Table 2). These findings are similar to those found by Atlasi et al. [3]. Levels of statistical significance were recorded for the AL width in the right cerebral hemisphere of males and females, as well as for the PL width in both cerebral hemispheres of males and females. The right anterior insula is involved in interoceptive awareness of homeostatic emotions including thirst, fatigue, pain, and heart rate. Increased accuracy in this subjective perception of the inner body is correlated with a larger amount of grey matter in the right anterior insular region [12]. The significant difference between males and females regarding the width of the right AL may be attributed to the general increased volume of grey matter in the right anterior insula and the fact that males tend to have anatomically larger and heavier brains compared to females [11, 12]. The differences in AL and PL widths between males and females may be attributed to the difference in brain sizes as well as the functional associations of each insular region.

Intra-observer agreement was fair to almost perfect for assessments regarding insular morphology (Table 3). The intra-observer reliability coefficients indicated that the morphometric parameters were recorded in a reliable way (Table 4).

To the best of the authors’ knowledge, no previous study has analysed the widths of the insular gyri using MRI scans. Therefore, this study plays an integral role in contributing to the current body of anatomical knowledge on the insula by providing morphological and morphometric data from a radiological perspective. The findings may aid clinicians in the diagnosis and treatment of insula-related abnormalities.

A limitation of this study may include the potential presence of degree of error due to the curvature of the insular cortex as well as the nature of the MRI and DICOM viewer software. Future studies may opt to include scans where all gyri are visible. Measurements may be more accurate if multiple MRI slices are used to reconstruct the insula as it will allow for a more comprehensive analysis of insular structures. Additionally, the use of a common criterion for classification is recommended to ensure consistency. The accessory gyrus and Heschl gyrus were excluded from this study as their presence was inconsistent and often difficult to accurately visualize due to the curved nature of the insula. The transverse gyrus was also excluded as it is best visualized from an inferior view, while this study used sagittal MRI scans only. Future studies may consider utilizing axial imaging to analyse the transverse gyrus. Additionally, demographical information such as age and race was not available for analysis and thus is a limitation of the study. The handedness of individuals was not considered in this study as that information was not available. Future research may also investigate the association of handedness with insular anatomy.

## Conclusion

This study confirmed that MR imaging modalities may be used to accurately represent the anatomy of the insula. The insula is predominantly trapezoidal in shape. The CIS, ASG, PSG, and ALG were predominantly “well seen”, while the PLG was comparably variable between “well seen” and “poorly seen”, and the MSG was more variable in visibility, being “not seen” more often than the other gyri and being “well seen” less often. Visibility of the MSG is dependent on sex but not on laterality. As the CIS was almost always clearly visible, it may be considered to be a reliable anatomical landmark on MRI. The PLG presented with the smallest mean widths. The mean values for the insular gyri and lobule widths were generally larger in males than in females. It is also likely that the smaller mean widths of the MSG attribute to its variation in visibility. The morphological and morphometric data obtained in this study may assist neurosurgeons to visualize the insula on MRI and may aid in preoperative planning for safer approaches during insula-related surgical procedures.

## Data Availability

No datasets were generated or analysed during the current study.
